# Preliminary study of online machine translation use of nursing literature: quality evaluation and perceived usability

**DOI:** 10.1186/1756-0500-5-635

**Published:** 2012-11-14

**Authors:** Ryoko Anazawa, Hirono Ishikawa, MJ Park, Takahiro Kiuchi

**Affiliations:** 1 Department of Social Medicine, Graduate School of Medicine, The University of Tokyo, Tokyo, Japan

**Keywords:** Online machine translation, Evaluation, Usability, Nursing literature, Japanese nurses

## Abstract

**Background:**

Japanese nurses are increasingly required to read published international research in clinical, educational, and research settings. Language barriers are a significant obstacle, and online machine translation (MT) is a tool that can be used to address this issue. We examined the quality of Google Translate® (English to Japanese and Korean to Japanese), which is a representative online MT, using a previously verified evaluation method. We also examined the perceived usability and current use of online MT among Japanese nurses.

**Findings:**

Randomly selected nursing abstracts were translated and then evaluated for intelligibility and usability by 28 participants, including assistants and research associates from nursing universities throughout Japan. They answered a questionnaire about their online MT use. From simple comparison of mean scores between two language pairs, translation quality was significantly better, with respect to both intelligibility and usability, for Korean-Japanese than for English-Japanese. Most respondents perceived a language barrier. Online MT had been used by 61% of the respondents and was perceived as not useful enough.

**Conclusion:**

Nursing articles translated from Korean into Japanese by an online MT system could be read at an acceptable level of comprehension, but the same could not be said for English-Japanese translations. Respondents with experience using online MT used it largely to grasp the overall meanings of the original text. Enrichment in technical terms appeared to be the key to better usability. Users will be better able to use MT outputs if they improve their foreign language proficiency as much as possible. Further research is being conducted with a larger sample size and detailed analysis.

## Findings

### Background

Japanese nurses are increasingly required to have knowledge of the research literature published internationally that is relevant to their clinical practices, education, and research activities [1,2]. To identify and acquire scientific evidence for their practice as well as to write up their own nursing research, nurses need to be able to understand nursing-related literature and to use technology efficiently [3]. However, the language barrier can be a significant obstacle for Japanese nurses, who need tools that can assist them to better understand nursing articles written in foreign languages. Use of technology such as machine translation (MT) may be an important step in this direction [4].

MT is a system in which text in one language is automatically translated into another language [5]. Therefore, it is a useful tool that assists multilingual communication. To date, a variety of MT approaches have been studied and widely used. However, before the era of the Internet, MT was too expensive for the general population and was limited to specific users [5]. In recent years, online MT systems have become widely available over the Internet [6-8], enabling a vast number of people to freely and readily access multilingual translations. These MT systems have been traditionally evaluated by both manual and automatic methods. In manual evaluation, each sentence or paragraph is evaluated by a human being according to the criteria of adequacy and fluency, usually using a five-point Likert scale [9]. In contrast, in most automatic evaluations of MT systems, statistical algorithms are used to measure the degree of similarity between the translation output and one or more reference translations [10]. Automatic evaluation has the advantage of reduced human cost; however, the actual meaning of the translation is indeed a critical component that can be resolved only by human evaluation [11].

In our previous study [4], we tested an existing manual evaluation method [12,13] for determining the quality of translations of nursing research articles from English into Japanese using four different online MT systems. This evaluation method, originally for English-Japanese pharmaceutical literature for medical doctors, used the criteria of structural accuracy and intelligibility of translated sentences, and the reliability of the method was verified. Also, in this previous study, Google Translate® (GT) was identified as a possibly usable MT system among the major online systems provided within Japan. The performance of GT had been similarly tested in several previous studies and was reported to outperform other systems [14-17]. Therefore, translations from GT were used in this preliminary study using a small sample of the Japanese nursing population to evaluate the quality of translation and the perceived usability of online MT. We also assessed another language pair, Korean into Japanese, to better determine the potential use of MT for the Japanese nursing population. Nursing exchanges between Japan and its neighboring country South Korea have increased markedly in recent years in all clinical, education, and research fields. Hence, accessing nursing information written in Korean might be an important option for Japanese nurses in the future.

The framework of this study is based on nursing informatics in health communication. In health communication, the transmission of information among health professionals is a significant factor in improving health care delivery. Nursing informatics applies information technology to the healthcare skills and tasks of nurses. It deals with the literacy of nurses in computer science and informatics for communicating data, information, and knowledge of nursing practice [18]. If nurses want to read nursing articles in an unfamiliar foreign language, they are hindered from utilizing relevant research and are thereby deprived of an opportunity to extend their knowledge. MT technology may contribute to solving this problem of language barrier by providing nurses in non-English speaking countries the additional motivation of being able to read technical literature with fewer burdens, improving their clinical, educational, and research practices and eventually leading to greater patient satisfaction. This study attempts to examine and discuss the feasibility of online MT technology use by nursing professionals to obtain technical information from articles written in an unfamiliar foreign language. In this preliminary study, we asked the following questions: How are translations from GT rated by Japanese nurses? How do Japanese nurses perceive the usability of online MT systems? In the sections that follow, we present a succinct report on the results of the study and discuss issues to pursue in future work.

### Methods

#### Preparation of study materials

For each of the language pairs, English–Japanese and Korean–Japanese, 141 sections of 23 randomly selected nursing study abstracts were translated using GT. The section types included Title, Aim/Background, Method, Results, Conclusions/Discussion, and Implications for Clinical Practice, in accordance with the structured abstract form generally employed in nursing journals. The number of sections in an abstract varied from five to seven, depending on the study and the journal rules (e.g., some abstracts may not have had the Discussion or Implications for Clinical Practice sections). Each section contained one to three sentences or phrases. The abstract sections used were from prominent nursing journals, such as *Journal of Nursing Research, Journal of Advanced Nursing,* and *Journal of Clinical Nursing*, which are accessible free of charge [4,19]. The subject areas of the nursing articles (cancer nursing, psychiatric and mental health nursing, maternal/child nursing, geriatric nursing, and chronic illness nursing) were selected according to papers presented at the annual convention of the Japan Academy of Nursing Science, as it is presumed that these areas are of major interest among the Japanese nursing population [4,19]. The source nursing abstracts needed to be consistent in the English and Korean languages for common understanding of the contexts in both languages; therefore, the Korean abstracts used in this study were translations from English. The English abstracts were translated into natural Korean expressions by a Korean speaker who is a nurse researcher, and is also trilingual in Korean, Japanese, and English. These Korean translations were then further validated by a Japanese professional medical translator with skill in English–Korean–Japanese translation, who checked for omissions and equivalencies. Thus, the naturalness and authenticity of the Korean versions of the abstract texts have been secured.

#### MT evaluation measurements

In our previous study, which employed a manual method of MT quality evaluation using the criteria of structural accuracy and intelligibility, the reliability of the evaluation method was verified [4]. In the current study, each section of the source abstract was evaluated with the criterion of intelligibility, as in our previous study [4], while a criterion of perceived usability, as in a previous study of general users [20], was used to evaluate the translation quality of the whole abstract. The operational definition of intelligibility used in previous studies was the extent to which the translations were understood and the accuracy of the information transmitted [12,21]. As usability has not been previously defined, it was operationally defined in this study as the extent to which the users felt that the translations were helpful in their grasping the meaning of the original text. A five-point Likert scale was used for criteria, as shown in Table 1. The nursing abstracts in their source language were not presented to the participants because of potential bias, depending on individuals’ degree of knowledge of the source language.

**Table 1:** Evaluation criteria for intelligibility and usability

**Scale**	Intelligibility *	Usability **
1	Not intelligible at all	Not usable at all
2	Only partially intelligible	Not usable so much
3	Somewhat intelligible, but not sure	Neither usable nor unusable
4	Almost intelligible	Usable in some degree
5	Everything is very intelligible	Very usable

Respondents were asked the following.

* “Please indicate the intelligibility of the translations with the criteria.

** “About the translated abstract as a whole: Do you feel that it is usable to grasp the meanings of original sentences.

#### Online MT system

GT was evaluated for the intelligibility and usability of its translations.

#### Survey questionnaire

A survey questionnaire that included two major parts was generated. One part evaluated the quality of the translations from GT, and the other part considered the respondents’ attributes, with questions related to online MT use.

#### Participants

We aimed to select a study population of native Japanese speakers with some nursing experience to understand clinical contexts in technical papers. Assistants and research associates^a^ at nursing universities were assumed to be appropriate. Fifty candidates from nursing universities throughout Japan were randomly chosen and the survey was distributed. A total of 28 individuals responded to the survey, a response rate of 56%. As there were 23 kinds of nursing abstract, each was distributed to two or three individuals for translation evaluation.

#### Collection of data

Each participant completed an evaluation questionnaire about the translations from GT, as well as a questionnaire about his/her respective demographic background, frequency of reading nursing literature in foreign languages, frequency of experiencing a language barrier when reading foreign nursing literature, and experience using online MT systems and their perceived usability. Additional file 1 and Additional file 2 show the questionnaire forms which were answered by the participants in their native language, Japanese. The surveys were distributed and returned by mail. The data were collected during February and March, 2012.

#### Analysis of data

The quantitative data were analyzed using the Statistical Package for the Social Sciences (SPSS) version 20. Data analysis was conducted using descriptive statistics, *t-*test, and text analysis.

### Ethical review

The Ethical Review Board of the University of Tokyo approved this study.

### Results

#### Demographic data

Respondents to the survey included 25 (89%) women and 3 (11%) men. Respondents’ age groups were as follows: 20s (n = 5, 18%), 30s (n = 16, 57%), 40s (n = 6, 21%), 50s (n = 1, 4%). Concerning job title, 19 (68%) of the respondents were research associates, and 9 (32%) were assistants. Academic degrees held by the respondents included a master’s degree (n = 16, 57%), bachelor’s degree (n = 10, 36%), and associate degree (n = 2, 7%). The average number of years of clinical and teaching professional experience was 7.93 and 3.96, respectively.

#### Evaluation of online MT quality

Table 2 presents the evaluation results. A total of 169 and 174 sections were evaluated for intelligibility of the translations in the language pairs of English-Japanese and Korean-Japanese, respectively. Usability was evaluated for the translation of the entire abstract as a whole, and 27 abstracts were evaluated for each language pair. When the mean scores were simply compared between English-Japanese and Korean-Japanese, translation performance was significantly better in Korean-Japanese with respect to both intelligibility and usability. Also, as shown in Table 3, the mean scores of intelligibility for each abstract section was lowest for the translations of “Results” in both language pairs (English-Japanese: 2.14; Korean-Japanese: 2.96).

**Table 2:** Evaluation of translation of English into Japanese, and Korean into Japanese

	**English into Japanese**	**Korean into Japanese**	***t***	***P***
Intelligibility Mean (SD)	n* = 169	n* = 174	8.046	<0.001
	2.46 (0.99)	3.38 (1.14)		
Usability Mean (SD)	n** = 27	n** = 27	3.189	0.002
	2.59 (1.19)	3.59 (1.12)		

n* the total number of unit sections for translation evaluation, according to structured abstracts, e.g., “Title” “Aim” “Background” “Method” “Results” “Discussion” and

“Conclusions”.

n** the total number of whole abstracts evaluated for usability of the translation.

**Table 3:** Evaluation scores according to abstract sections

**Average evaluation score of translations (SD) for abstract sections**								
	**Title**	**Background**	**Purpose**	**Method**	**Results**	**Discussion**	**Conclusion**	**Relevance to clinical practice**
English-Japanese *n*	2.41 (0.80)	2.44 (0.96)	2.36 (0.99)	2.82 (1.16)	2.14 (0.89)	2.22 (0.83)	2.67 (1.19)	2.67 (0.82)
	27	25	28	28	28	9	18	6
Korean-Japanese *n*	3.63 (1.28)	3.57 (1.00)	3.33 (1.14)	3.46 (1.14)	2.96 (1.07)	3.80 (1.10)	3.17 (1.17)	3.43(0.98)
	27	28	27	28	28	5	24	7

#### Frequency of reading nursing literature in English and perceived language barrier

When asked to report the number of nursing articles that they had read in English, more than half of the respondents answered one to five articles. Eighteen percent had read more than six articles. One third of the respondents had not read any (Table 4). Also, 22 out of 24 respondents perceived a language barrier often or always when they read nursing literature in English (Table 3).

**Table 4:** Number of nursing articles read in a foreign language (English) within approximately 3 months and perceived frequency of experiencing a language barrier

	**None**	**1 to 5**	**6 to 10**	**11 to 20**	**More than 21**
Number of nursing articles read in English (*n*=27)	8 (30%)	15 (55%)	4 (14%)	1 (4%)	0 (0%)
	**Never**	**Rarely**	**Sometimes**	**Often**	**Always**
Perceived frequency of language barrier (*n*=24)	1 (1%)	0 (0%)	1 (1%)	9 (32%)	13 (46%)

#### Current online MT use and perceived usability by participants

**Figure 1:**
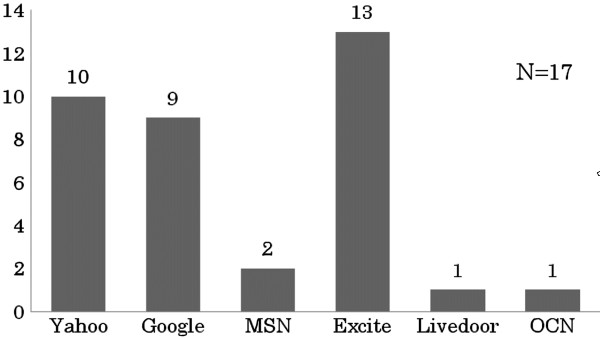
Number of respondents who used online MT systems provided by internet retrieval engines (multiple answers permitted).

Of 28 participants, 17 (61%) had experience using an online MT system. Figure 1 shows the major Internet retrieval engines that provide online MT systems used by the participants. Excite® was the most popular system among the study participants. The only language pair that the participants had experienced with online MT systems was English-Japanese. In answer to the question asking with what frequency they had used online MT systems, one (6%) respondent answered “always,” six (35%) answered “sometimes,” six (35%) answered “often,” and four (24%) answered “do not use any more.” When asked about the usability of online MT systems in general, 10 (59%) respondents answered that the system is “not very usable” and 7 (41%) answered that it is “usable to some degree.”

The participants were able to comment on their perceptions of usability and their impressions of online MT systems. Written comments were obtained from 17 respondents, and these data were categorized into the following major themes: 1) online MT use and usability, 2) participants’ own English proficiency, and 3) participants’ expectations for online MT systems. Several elements were identified for each of these themes. Concerning theme 1, comments included “improper translations of technical terms by online MT system,” “difficulty in translating contexts by online MT systems,” “no use due to improper translations,” “use only for grasping the meaning of source sentences,” and “use only for checking single words.” Concerning theme 2, comments included “limited English proficiency” and “need to improve English ability.” For theme 3, the comment “enriched technical terminologies in online MT systems” was received. The typical comments are shown in below:

– Always feel the limitation of MT. Translating medical and nursing terminologies by online MT is difficult to do. I have to have enough ability in the English language as a researcher.

– [When using MT] I would need to validate the translations from MT to correctly understand the article after all. Online MT would be useful only on the occasion in which I am looking for any literature related to my own research theme.

– I have never used online MT due to its image of producing inaccurate translation outputs, but it would be great if the tool were usable for a person like me who is not good at reading in a foreign language.

– Online MT is useful only for translating words, not for sentences.

– Authentic online MT performance is expected.

– Reading the translation from online MT is exhausting. They are not intelligible at all. I am not good at English, but I recognize that I need training in English.

– It would be helpful if there were good online MT system for Japanese-to-English translation.

– The problem is that online MT systems make errors in medical and technical terminology. Improvement in the nursing domain is expected.

### Discussion

The evaluated mean scores for intelligibility and usability in English-Japanese translations were both less than three on a five-point scale, indicating insufficient performance for users under both criteria.

For both intelligibility and usability, the evaluation scores were significantly higher for translations from Korean into Japanese than for English into Japanese. Language structure, such as word order, significantly influences the quality of translations [22]. The word order of the Japanese language is similar to that of Korean, and many common vocabularies exist between these two languages, whereas there are major structural differences between Japanese and English. Therefore, there are limitations to how directly one can compare the translation quality between these languages pairs using the mean values. In this small sample size of study participants, no one had read a technical paper in the Korean language, but our study findings suggest that GT could be a relatively practical tool for Japanese nurses to read Korean articles.

Translations tended to be rated low in the “Results” section of the abstracts. In this section, most of original texts were composed of complex sentences containing extensive information.

When the sentence to be translated is long, the performance of translation tends to be poor [12,13]. Also, current MTs do not consider context [11], which limits their usability for reading unitary sentences with meanings. The mediation of word count and complexity of the source sentences requires further investigation.

Our preliminary study found that approximately 60% of the respondents had used online MT systems, and nearly 60% said they were not very useful. Considering the results above, current online MT systems are not practical enough for full-scale use with the nursing literature.

Important indications were gained from the comments made by the respondents. Japanese nurses perceive online MT systems as lacking technical term vocabularies, they tend to use this tool to roughly understand the meaning of the whole text, and they felt the need to improve their own language proficiency. These remarks suggest that the performance of online MT systems could be improved by considering the way Japanese nurses use the systems and what they expect. At the same time, how to constructively use MTs ought to be included as part of the foreign language education of primary nursing students.

The importance of the English language to their practice and research is recognized by the Japanese nursing population. Although our study showed the number of English articles read by the respondents, we cannot directly know from these numbers if they read enough or not enough due to lack of language proficiency. However, it can be fairly assumed that the language barrier is an obstacle for them to read articles in English. Given that the quality of translation output is not entirely reliable, a certain level of foreign language proficiency is required to use MT systems successfully [23]. It is difficult to comprehend the output from an MT system without knowledge of the language structure, especially for language pairs such as English-Japanese, where the basic grammatical structures are very different. When users’ English language proficiency is adequate but not excellent, they may be able to compare the source text and the MT output to determine whether the translations are usable without naively accepting them. Considering the current performance limitations of English–Japanese MTs, it is necessary for users to improve their own English proficiency for improved utilization of MT systems.

The results of this study suggest the possibility of encouraging multilingual nursing exchanges in the future, including hyperlinks to online multilingual translations on the websites of nursing journals. Also, the importance of English language education for nurses is underlined, especially in the context of continuing education in the workplace.

### Conclusion and future work

In this preliminary study, the quality of English-Japanese and Korean-Japanese translations of nursing literature using GT was evaluated by assessing the intelligibility and usability of the translations to a small sample size of the Japanese nursing population. The study participants were asked about their experience and perception of online MT systems. The results implied that nursing articles translated from Korean into Japanese could be read through an online MT system of GT at an acceptable level of comprehension, but the MT was not sufficient for English-Japanese translations. Respondents with experience using MTs use this tool largely to grasp the overall meaning of the original text. Enrichment in technical terms in the online MT system appeared to be the key to better usability.

Although an MT system is currently the tool of choice for lowering the language barrier, considering the grammatical structural differences between English and Japanese, users will be better able to use MT outputs if they improve their foreign language proficiency as much as possible.

Currently, further research is being conducted with a larger sample size. It is expected that this research will explore the factors associated with the evaluation of translation quality of GT, and will elucidate the relationship between study participants’ attributes and their online MT use with multiple variance analysis to lead to a discussion of how to better utilize this technological tool for the nursing population.

## Endnotes

^a^In Japanese academia, faculty members with the titles of “assistant” and “research associate” are those who are in the positions of “assisting teaching and research” and “conducting teaching and research,” respectively. Nevertheless, required qualifications for hiring these nursing academic staff members and their actual duties are not always consistent among nursing universities across Japan. Commonly, 3–5 years of nursing experience is required before applying for these positions.

## Competing interests

The authors’ declare that they have no competing interests.

## Authors' contributions

AR, IH, KT designed the study. AR and P MJ prepared the source data. AR analyzed the data. HI gave statistical advises. AR, IH wrote the manuscript. All authors read and approved the final manuscript.

## Supplementary Material

Additional file 1 Questionnaire form for evaluation of translations by GT (in Japanese). Click here for file

Additional file 2 Questionnaire form for participants’ demographic background, frequency of reading nursing literature in foreign languages, frequency of experiencing a language barrier, and online MT use and perceived usability (in Japanese). Click here for file
