# Supplementary material for: Preliminary study of online machine translation use of nursing literature: quality evaluation and perceived usability
Source: BMC Res Notes. 2012 Nov 14;5:635. doi: 10.1186/1756-0500-5-635 (PMC3576310; doi:10.1186/1756-0500-5-635)
Supplement: Additional file 1 — Questionnaire form for evaluation of translations by GT (in Japanese). [file 1756-0500-5-635-S1.docx]

**翻訳された日本語文を以下の「理解度の評価基準」に沿って評価してください**

訳出された日本語訳 １. 全体的にまったく理解できない

２. 部分的に理解可能な箇所はあるが、全体としては理解できない

３. 全体的になんとなく理解はできるが、理解に自信がもてない

４. 全体がほぼ、良く理解できる

５. まったく問題なく、すべて良く理解できる

| **日本語へ翻訳した文** | **理解度の評価基準** | | | | |
| --- | --- | --- | --- | --- | --- |
| 1（タイトル）女性の仕事の能力上の化学療法による認知の副作用の認識：フォーカスグループの調査。 | 1 | 2 | 3 | 4 | 5 |
| 2（目的）化学療法誘発性認知の変化、意思決定や作業能力の認識を動作するように日々のタスクや後続の戻りを実施する上で認知限界の彼らの認識の女性の意識を調査する。 | 1 | 2 | 3 | 4 | 5 |
| 3（背景）　証拠は、女性は化学療法の治療の結果として、乳がんの経験認知の変化と診断されたことを示唆している。これらの変更は、メモリ、濃度と情報を整理する能力の微妙な赤字になりがちですが、彼らは患者の作業能力と、その後の雇用の決定に影響を与えることができる方法の識別も発表された研究は存在しなかった。 | 1 | 2 | 3 | 4 | 5 |
| 4（方法）　これは、定性的な研究であった。 データは2つのフォーカスグループ（N = 6、N = 7）と半構造化面接を用いて乳癌の生存者から収集した。インタビューは逐語的に転写され、テンプレートの解析を用いて分析した。 | 1 | 2 | 3 | 4 | 5 |
| 5（結果）　データは4つの主要なテーマに分類した： （1）中の認知の変化の認識とは、次の化学療法、（2）認知能力と引き換えに信頼が機能するために、  （3）仕事の能力上の認知の変化の影響と化学療法の認知の副作用について（4）情報。職場復帰とその後の仕事の能力に向かってビューと乳癌の生存者の経験は、化学療法誘発性認知障害の影響を受けました。具体的には仕事や作業を管理する能力に戻っての評価は、3つの相互に関連の要因によって影響された。 （1）実際の認知能力は、次の化学療法、（2）女性とその家族による認知障害の認識と仕事のタスクを含む日常業務を遂行の自信（3）その後の影響。 | 1 | 2 | 3 | 4 | 5 |

次ページへ　☞

| **日本語へ翻訳した文** | **理解度の評価基準** | | | | |
| --- | --- | --- | --- | --- | --- |
| 6（結論）　より多くの情報およびサポートは、がんの患者さんが自宅と職場での化学療法誘発性認知障害を管理するために必要とされる。看護師は、ますます癌との仕事上でその治療の影響について質問されているので、よくこの助言を提供するように配置されています。その後、看護師は、追加の知識と指導は、この情報とサポートを提供する必要があります。 | 1 | 2 | 3 | 4 | 5 |

|  |
| --- |

「翻訳文１」全体について、あてはまる数字に○をつけて下さい。

この翻訳文全体は、原文の意味を把握するのに役立ったと感じますか

１）まったく役に立たない

次ページへ　☞

２）あまり役に立たない

３）どちらともいえない

４）少しは役に立つ

５）かなり役に立つ

次ページへ　☞

次ページへ　☞

　訳出された日本語訳を１. 全体的にまったく理解できない

**翻訳された日本語文を以下の「理解度の評価基準」に沿って評価してください**

２. 部分的に理解可能な箇所はあるが、全体としては理解できない

３. 全体的になんとなく理解はできるが、理解に自信がもてない

４. 全体がほぼ、良く理解できる

５. まったく問題なく、すべて良く理解できる

翻訳文２（看護学論文の抄録１件分）

次ページへ　☞

| **日本語へ翻訳した文** | **理解度の評価基準** | | | | |
| --- | --- | --- | --- | --- | --- |
| 1（タイトル）軽度外傷の緊急治療室で治療を受ける患者の精神障害。 | 1 | 2 | 3 | 4 | 5 |
| 2（背景）　救急部（ED）の全患者の35％は身体的外傷と来る。 | 1 | 2 | 3 | 4 | 5 |
| 3（目的）　Axis I / IIの精神障害の既往歴や現病歴があり、身体的外傷でEDを受診する患者の割合を調査し、精神疾患の既往歴のある患者、精神疾患の病歴がない患者は、現在の精神障害がある患者を比較するものとする。 | 1 | 2 | 3 | 4 | 5 |
| 4（方法）　緊急治療室の患者中、解剖学的な外傷は認められるが、生理学的に正常人275人を無作為に選出した。除外基準は：過去2年間の外傷、内科的疾患や家庭内暴力による外傷、あるいは重症のうつ病や精神疾患の治療についてのレポート。精神科の病歴や現在の病気は、精神障害の診断と統計の手引きの構造化臨床面接、第4版（DSM - IV）（構造化精神科面接）を用いて診断された。カイ二乗の変量解析を用いて3群（精神科の病歴があること、精神科の病歴なし、現在の精神疾患がある）を比較した。 | 1 | 2 | 3 | 4 | 5 |
| 5（結果）　サンプルには57.1％黒人と39.6％、白人の、男性（51.6％）、女性（48.4％）が含まれていた。このセムプルジュン103人の患者（44.7％）が、精神科の病歴（n = 80）、または現在の精神科疾患（n = 43）のDSM - IVの基準に合った。うつ病の既往歴（24％）は、その他の既往歴が発生することがより多かった（不安の6％、アルコール飲用/乱用の14％、薬物の使用/乱用の15％、適応23％、行動障害の14％）。現在の気分障害（47％）も、その他の現在の診断よりも多かった（不安10％、アルコール16％、薬物の7％、適応7％、人格障害12％）。現在、精神疾患の診断を受けた患者は、外傷をしたときに、失業中であった傾向が顕著だ（p。001）。 | 1 | 2 | 3 | 4 | 5 |
| 6（結論）　深刻な外傷を受けた患者さん個人々では多くの精神科的合併症や精神科の病歴が見えた。計画的な医療制度で身体的外傷の治療のために連絡することは（特にED）看護師としてのにとって精神疾患の患者を確認し、患者に適切な治療を言及するようにする機会を提供する。 | 1 | 2 | 3 | 4 | 5 |

次ページへ　☞

「翻訳文2」全体について、あてはまる数字に○をつけて下さい。

この翻訳文全体は、原文の意味を把握するのに役立ったと感じますか

１）まったく役に立たない

２）あまり役に立たない

３）どちらともいえない

４）少しは役に立つ

５）かなり役に立つ

次ページへ　☞

次ページへ　☞

調査票への記入は以上です。

○○先生のご協力に心より感謝申し上げます。
