# Supplementary material for: Preliminary study of online machine translation use of nursing literature: quality evaluation and perceived usability
Source: BMC Res Notes. 2012 Nov 14;5:635. doi: 10.1186/1756-0500-5-635 (PMC3576310; doi:10.1186/1756-0500-5-635)
Supplement: Additional file 2 — Questionnaire form for participants’ demographic background, frequency of reading nursing literature in foreign languages, frequency of experiencing a language barrier, and online MT use and perceived usability (in Japanese). [file 1756-0500-5-635-S2.docx]

属性とオンライン機械翻訳の利用に関する以下の質問にお答えください。

質問１．　各項目のあてはまるものに○をつけるかご記入ください

・性別　１）女性　　２）男性

・年齢　１）20代　２）30代　　３）40代　４）50代　５）60代

　・職位　１）助手　　２）助教

　・学位　１）専門士　２）準学士　３）学士　　４）修士　　５）博士

　・臨床経験年数　［　　　　　］年　・教員経験年数 [ ]年

・語学力に関する資格試験のスコアなどがお分かりでしたらご記入ください

[英語]

TOEIC [　　　　　]点　TOEFL [　　　　　]点　英検　[　　　　　]級

IELTS　[　　　　　]点　　その他　[　　　　　　　　]点、級、レベル

　[英語以外の外国語]

（　　　　　　）語、試験名（　　　　　　　　　　　）：[　　　　]点、級、レベル

（　　　　　　）語、試験名（　　　　　　　　　　　）：[　　　　]点、級、レベル

質問２．　最近の３ヶ月間位で、ご自身の専門領域に関連する**外国語の**文献を何本程度読みましたか。以下の本数の中からあてはまるものを選び、番号に○をつけてください。

| 言語 | | 本数 |
| --- | --- | --- |
| 英語文献 | | 1. 0本 ②１～5本　③ 6～10本　④ 11～20本   ⑤ 21本以上 |
| 英語以外の  外国語の文献を読んだ場合 | （　　　　　　語） | 1. １～5本　② 6～10本　③ 11～20本   ④ 21本以上 |
|  | （　　　　　　語） | 1. １～5本　② 6～10本　③ 11～20本   ④ 21本以上 |

次ページへ

質問３．　専門領域の外国語文献を読む際、どの程度の頻度で言葉の壁を感じますか。英語文献と、英語以外の文献を読まれる場合はその言語について、以下の数字からあてはまるものを選び、番号に○をつけてください。

| 言語 | | 言葉の壁を感じる頻度 |
| --- | --- | --- |
| 英語文献 | | ① 感じたことは一切ない　② めったに感じない  ③ ときどき感じる　④ 頻繁に感じる　⑤ 常に感じる |
| 英語以外の  外国語の論文を読む場合 | （　　　　　語） | ① 感じたことは一切ない　② めったに感じない  ③ ときどき感じる　④ 頻繁に感じる　⑤ 常に感じる |
|  | （　　　　　語） | ① 感じたことは一切ない　② めったに感じない  ③ ときどき感じる　④ 頻繁に感じる　⑤ 常に感じる |

質問４．　専門領域の外国語文献を読む際に、オンライン機械翻訳を利用したことがありますか。あてはまる番号に○をつけてください。

　１．利用したことは無い（下記の１．の質問にお答えください）

　２．利用したことがある（下記の２．の質問にお答えください）

１．「利用したことは無い」とお答えの場合：その理由について、あてはまるものに○をつけてください

①必要ないから

②役に立たないと思うから

③オンライン機械翻訳の存在を知らなかった

④その他の理由　[　　　　　　　　　　　　　　　　　　　　　　　　　]

２．「利用したことがある」とお答えの場合：以下１）～４）の質問に対して、あてはまるものに○をつけるかご記入ください。

１）ご利用の頻度はどの程度ですか

①常に/ほぼ常に利用する　②ときどき利用する　③ごくたまに利用する

④現在は利用していない　→　理由：I 必要ないから　ii 役に立たないと思うから

iiiその他の理由　[　　　　　　　　　　　　　　　　]

２）どの検索エンジンの機械翻訳を利用されたことがありますか（複数回答可）。

① ヤフー　② グーグル　③ MSN (Bing)　④ エキサイト　⑤ ニフティ

⑥ ライブドア　⑦ OCN　⑧ So-net　⑨ その他　[　　　　　　　　　　　]

⑩ どの検索エンジンか覚えていない

次ページへ

3）どの言語の組み合わせで利用されましたか（複数回答可）

　　①英語から日本語

②英語以外の外国語から日本語：[　　　　　　]語、[　　　　　　]語

4）機械翻訳をご利用になった感想はいかがでしたか。英語と、英語以外の外国語（ご使用経験がある場合）に関して、以下の感想の中から、ご自身が経験した言語に関して選び、番号に○をつけてください。

| 言語 | | 感想 |
| --- | --- | --- |
| 英語→日本語 | | ①かなり役に立つと感じた  ②少しは役に立つと感じた  ③どちらともいえない  ④あまり役に立たないと感じた  ⑤まったく役に立たないと感じた |
| 英語以外の  外国語→  日本語 | （　　　　　語） | ①かなり役に立つと感じた  ②少しは役に立つと感じた  ③どちらともいえない  ④あまり役に立たないと感じた  ⑤まったく役に立たないと感じた |
|  | （　　　　　語） | ①かなり役に立つと感じた  ②少しは役に立つと感じた  ③どちらともいえない  ④あまり役に立たないと感じた  ⑤まったく役に立たないと感じた |

◆機械翻訳利用に関するその他の感想や、全体を通して何かコメントなどございましたらご記入ください。

質問票への記入は以上で終了です。ご協力に心より感謝いたします。
